# Antiseizure medication and SUDEP – a need for unifying methodology in research

**DOI:** 10.3389/fneur.2024.1385468

**Published:** 2024-04-17

**Authors:** Dag Bruheim Aurlien, Erik Taubøll

**Affiliations:** ^1^Neuroscience Research Group and Department of Neurology, Stavanger University Hospital, Stavanger, Norway; ^2^Department of Neurology, Oslo University Hospital, Oslo, Norway; ^3^Faculty of Medicine, University of Oslo, Oslo, Norway

**Keywords:** antiseizure medication (ASM), SUDEP, carbamazepine, lamotrigine, IGE (idiopathic generalized epilepsy), females

## Abstract

The risk of sudden unexpected death in epilepsy (SUDEP) increases with the frequency of generalized tonic–clonic seizures. Carbamazepine (CBZ) and lamotrigine (LTG) have been suggested to increase the risk. However, the prevailing viewpoint is that the choice of antiseizure medication (ASM) does not influence the occurrence. We have explored the approach to addressing this question in relevant studies to evaluate the validity of the conclusions reached. A systematic search was performed in PubMed to identify all controlled studies on SUDEP risk in individuals on CBZ or LTG. Studies were categorized according to whether idiopathic generalized epilepsy (IGE) or females were considered separately, and whether data were adjusted for seizure frequency. Eight studies on CBZ and six studies on LTG were identified. For CBZ, one study showed a significantly increased risk of SUDEP without adjustment for seizure frequency. Another study found significantly increased risk after statistical adjustment for seizure frequency and one study found increased risk with high blood levels. Five other studies found no increase in risk. For LTG, one study showed a significantly increased risk in patients with IGE as opposed to focal epilepsy, and another study showed a significantly increased risk in females. None of the subsequent studies on LTG and none of the studies on CBZ considered females with IGE separately. Taken together the available studies suggest that LTG, and possibly CBZ, may increase occurrence of SUDEP when used in females with IGE. Additional studies with sub-group analysis of females with IGE are needed.

## Introduction

Sudden unexpected death in epilepsy (SUDEP) primarily affects young adults and, among neurological diseases, only stroke causes more loss of life-years (1). In most observed documented cases, it has occurred in relation to a generalized tonic–clonic seizure (GTCS) (2). Typical GTCSs rarely occur in infants and toddlers and are characterized by a shorter tonic phase and by shorter postictal generalized EEG suppression (PGES) providing an explanation why SUDEP is so extremely rare among the youngest individuals with epilepsy (3). The incidence of SUDEP increases with the frequency of GTCSs and seizure control is probably the most effective preventive measure (4, 5). The highest incidence of SUDEP has been found in untreated patients (6) and the use of effective antiseizure medication (ASM) has resulted in the incidence of SUDEP being reduced by more than seven times (7).

The mechanisms underlying this devastating outcome of epilepsy are still incompletely understood. However, there is considerable evidence that central or obstructive apnea or ictal cardiac arrhythmia, alone or in combination with apnea, may be involved (8). In addition, some of these deaths may be explained by ictal electrocerebral shutdown, where persistent epileptiform activity in the electroencephalogram (EEG) is followed by PGES (9). Whether or not treatment with ASM can play a role in causing or triggering SUDEP and whether gender can play a role for the risk has been discussed for several years (10–14). To date, only two ASMs, carbamazepine (CBZ) and lamotrigine (LTG), have been suggested to be implicated. Still, the present prevailing opinion is that choice of ASM does not affect the risk of SUDEP. However, conclusions from studies investigating this question depend on the methods used, the variables taken into consideration, and the way in which the results have been interpreted. The aim of this review is to explore how relevant studies on this topic have approached the question and whether all relevant variables have been taken into consideration. By applying this critical evaluation of these studies, it will be easier to determine the validity of any conclusions that have been reached.

## Methods

A systematic search was performed in PubMed restricted to English language articles only.

The inclusion criteria were controlled studies that estimated the relative risk of SUDEP in individuals treated with CBZ or LTG as compared with other ASM. No studies fulfilling the inclusion criteria were excluded. The search for relevant studies was concluded October 5, 2023.

We used the search terms «lamotrigine sudden unexpected death epilepsy», «carbamazepine sudden unexpected death epilepsy» and «risk factors sudden unexpected death epilepsy».

**Figure 1:**
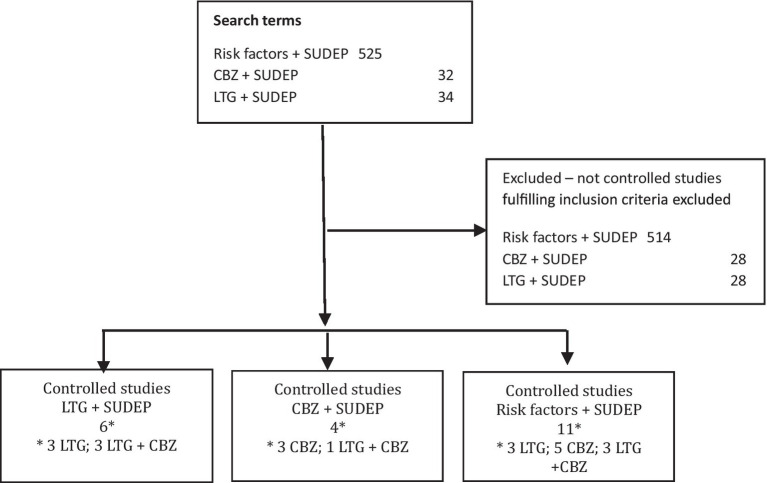
Flow diagram showing the search terms used, number of search results and selected studies. CBZ, carbamazepine; LTG, lamotrigine; SUDEP, sudden unexpected death in epilepsy.

The studies fulfilling the inclusion criteria (Figure 1) were categorized according to whether or not separate analyses were made for patients with idiopathic generalized epilepsy (IGE), whether analyses were divided by patient sex, and whether adjustment of results according to seizure frequency was performed.

## Results

All abstracts from the search results were read, and inclusion of studies fulfilling the inclusion criteria were based on full article texts. The search terms “lamotrigine sudden unexpected death epilepsy” provided 34 results and the search terms “carbamazepine sudden unexpected death epilepsy” provided 32 results of which six and four studies were selected, respectively. The search terms “risk factors sudden unexpected death epilepsy” provided 525 results of which 11 were selected. Only two of these were not identified by the two preceding searches. Five controlled studies fulfilled the inclusion criteria on CBZ, three on LTG, and three on both CBZ and LTG (Figure 1). Table 1 summarizes the selected information researched in the individual selected articles.

**Table 1:** Methods and conclusions in studies exploring a possible association between CBZ, LTG and SUDEP.

Study	Timmings (15)	Nilsson et al. (16)	Nilsson et al. (17)	Langan et al. (6)	Hitiris et al. (18)	Hesdorffer et al. (11)	Hesdorffer et al. (13)	Aurlien et al. (12)	Tomson et al. (14)	Sveinsson et al. (19)	Nightscales et al. (20)
ASM	CBZ	CBZ	CBZ	CBZ	CBZ	LTG	LTG and CBZ	LTG and CBZ	LTG	LTG and CBZ	LTG
Separate analysis for cases with IGE on LTG or CBZ	_	_	_	_	_	+	_	_	+	_	_
Separate analysis for female individuals on LTG or CBZ	_	_	_	_	_	_	+ (LTG)	+ (LTG and CBZ)	+	_	_
Separate analysis for female individuals with IGE on LTG or CBZ	_	_	_	_	_	_	_	_	_	_	_
Adjustment for seizure frequency	_^*^	+	+	+	_	_	+	_	NA **	+	+
Author’s conclusion on association with the ASM	Possible	No	Increased risk with high serum concentrationof CBZ. May be due to poor seizure control	Yes	No	Increased risk with LTG in IGE, not in focal epilepsy	No	Increased risk for females on LTG, but not for females on CBZ	No **	No	No

CBZ, carbamazepine; LTG, lamotrigine; SUDEP, sudden unexpected death in epilepsy; ^*^ Adjustment for seizure frequency not specified, but there was no difference in seizure frequency between cases and controls; ^**^ Wide confidence interval, an increased risk cannot be excluded.

For CBZ, a study from 1998 showed that treatment with this ASM was associated with a significantly increased risk of SUDEP without adjustment for seizure frequency (15) (Table 1). The majority of those who had died of SUDEP had IGE. A further study found an increased SUDEP risk in those treated with CBZ (6) and another study reported an elevated risk when serum concentrations of CBZ were high (17), with both these studies adjusting for seizure frequency. In contrast, two other studies found no increase in risk of SUDEP associated with CBZ treatment, with or without adjustment for seizure frequency (16, 18). Only one study analyzed females separately and this study found no increase in risk (12). None of the studies included a separate analysis for females with IGE.

For LTG, one study from 2011 showed a significantly increased risk of SUDEP in IGE as opposed to focal epilepsy (11) but did not analyze female gender separately. Another study from 2012 showed a significantly increased risk in females without analyzing females with IGE separately (12). The other four studies on LTG did not include separate analyses for IGE and/or female individuals and/or adjusted for seizure frequency; these studies concluded that there was no association between SUDEP and treatment with LTG (13, 14, 19, 20).

## Discussion

The majority of studies exploring a possible association between ASMs, specifically CBZ and LTG, and SUDEP have concluded that the type of ASM does not influence SUDEP risk (Table 1). Importantly, these studies only examined individuals with epilepsy at the group level and the conclusions were reached without a separate analysis for subgroups (13, 15, 16, 18–20). However, to our knowledge there is no evidence that indicates that the biological vulnerability to ASMs is the same in all individuals with a diagnosis of epilepsy. Indeed, as many epilepsies are caused by mutations in genes encoding for ion channels, and as the mode of action for many ASMs, including CBZ and LTG, is blocking of ion channels (21), there is a need to focus on the subgroups of individuals with IGE. In particular females with IGE are a significant subgroup. Study results have been the subject of considerable debate (10, 12–14). There are important reasons for this:

### ASM efficacy is crucial for the prevention of SUDEP, and CBZ and LTG may be inferior for treating cases of IGE

Discussion on whether particular ASMs could increase SUDEP risk was initiated by Timmings in 1998, when it was found that, among patients who had died of SUDEP, a significantly greater proportion (79%) used CBZ compared to controls (38%; *p* < 0.01) (15). A separate analysis for IGE was not performed, but a previous report (22) revealed that the majority of the deceased had IGE. There was no difference in seizure frequency between cases and controls. Nevertheless, several studies have documented that the sodium-channel blockers, including CBZ and LTG (21), are of lower efficacy when used in the treatment of IGE (23–25) and may even increase seizure frequency (26). Generally, if a treatment for a potentially lethal disorder has been shown to be less effective than other treatment options, then the argument that choosing the poorer treatment option could increase the risk of premature death is reasonable; the prescribing physician should be aware that this treatment could contribute to the cause of death. In studies comparing the efficacies of two different ASMs, usually only focal epilepsy or generalized epilepsy are included (23, 27, 28), as ASMs can work differently for treating focal epilepsy and generalized epilepsy. It is unclear why this principle is rarely applied in SUDEP research (6, 13, 15–20), and has, to our knowledge, never been explained in the literature.

Several studies on a possible association between SUDEP and treatment with CBZ and/or LTG have not included a separate analysis for IGE (6, 13, 15–20); they have concluded that there is no increase in SUDEP risk associated with these ASMs, and not made any distinction for the subgroup with IGE. In 2011, the largest study to date on ASM and SUDEP was published by Hesdorffer et al. (11) on commission from the International League Against Epilepsy (ILAE) Commission on Epidemiology; Subcommission on Mortality. This study, which included 289 SUDEP victims from four different countries, revealed a significantly increased risk of SUDEP for LTG users with IGE (odds ratio (OR) 2.2; confidence intervals (CI) 1.14–4.23), but not even a tendency towards an increased SUDEP risk for LTG users with focal epilepsy (OR 1.04; CI 0.57–1.92). The authors stated that independent studies were needed to “support or refute” whether LTG is a risk factor for SUDEP. However, only a few months later, the same authors published new analyses based on the same material and this time concluded that there is no connection between choice of ASM and SUDEP (13). Strangely, their contradictory findings from their previous study, indicating an increased risk of SUDEP when LTG was used in IGE patients only, were not discussed, and a separate analysis on the subgroup of IGE patients was not performed. Thus, it seems possible that dilution of their material, by mixing the smaller subgroup of patients previously shown to have an increased SUDEP risk (IGE), with a much larger subgroup with focal epilepsy without an increased risk, explains their finding of a lack of association of SUDEP with LTG treatment. Furthermore, the 2012 study (13) adjusted for differences in seizure frequency between cases and controls and concluded that increased SUDEP risk depends on the number of seizures, and not on the ASM. However, as seizure frequency itself depends upon the efficacy of the ASM, adjustment for seizure frequency thereby entails adjustment for differences in ASM efficacy. Adjusting for seizure frequency only answers the question of whether there is a difference between SUDEP victims and controls, independent of the GTCSs. The relevance of this issue is dubious, as SUDEP occurs in association with GTCSs (2), and it is unclear why this principle of a statistical correction, which implies adjustment for differences in ASM efficacy, has been so widely accepted (6, 13, 16, 17, 19, 20).

### In genetically predisposed individuals LTG, and possibly CBZ, may be associated with cardiac arrhythmia

Based on Timmings` (15) findings of a significantly higher proportion of SUDEP deaths in patients on CBZ compared to controls and no differences in seizure frequency in the 1998 study, the author suggested that one possible explanation could be a CBZ-induced elongation of the QT interval in the electrocardiogram (ECG), combined with a mild pro-arrhythmic effect of the seizures (15). CBZ has been associated with atrioventricular conduction delay, including potentially life-threatening bradyarrhythmia (29) and also with a slight, but significant, decrease in the QT interval in a study of elderly epilepsy patients (30). Furthermore, CBZ has been shown to reduce heart-rate variability in individuals with epilepsy (31). However, to our knowledge the arrhythmogenic potential of CBZ in IGE has not been explored in either clinical studies or *in vitro* studies.

In a publication from 2005, Danielsson et al. (32) demonstrated that LTG inhibits the cardiac rapid delayed rectifier potassium ion current (IKr). The authors suggested that by increasing the QT interval in the ECG, LTG could increase the risk of a fatal arrhythmia, possibly primarily in the presence of seizure-induced acidosis (32). The discussion of whether treatment with LTG could increase SUDEP risk in genetically predisposed individuals was initiated by a case series in 2007, where all four of the deceased were females with genetic epilepsy and all had been treated with LTG in monotherapy (10). Among possible explanations for this clinical observation, one suggestion was that LTG, when used in IGE, could increase the risk of cardiac arrhythmia, or provide inferior efficacy, or that a combination of these could have occurred (10). The following year, a study on LTG in clinically relevant doses in healthy subjects showed no increment of the QT interval, and the authors concluded that there is no convincing evidence that LTG is associated with cardiac arrhythmia (33). However, whether their finding of an apparent lack of association was due to the sole inclusion of healthy individuals (no individuals with IGE were included) was not considered. In 2009, postmortem sequencing of long QT syndrome (LQTS) genes from four SUDEP victims with idiopathic epilepsy (10) had revealed in one of the deceased a novel mutation (R523C) in the SCN5A gene that encodes the cardiac voltage-gated sodium channel, Nav1.5 (34). Mutations in this gene are associated with LQTS type 3 and Brugada syndrome (35). As the gene is co-expressed in heart and brain (36), it was suggested that this mutation could both explain the epilepsy and increase the probability of a pro-arrhythmic side effect of LTG.

Recently, a study used whole-cell voltage clamp electrophysiology to examine the influence of three rare genetic variants of the SCN5A gene found in SUDEP victims on channel function (37). The results revealed that these genetic variants significantly influenced Nav1.5 channel function, suggesting a potential to increase the risk of cardiac arrhythmia. Importantly, therapeutic concentrations of LTG also significantly changed the channel function and the authors suggested that LTG may increase the risk of cardiac arrhythmia in individuals carrying mutations in cardiac arrhythmia genes. This study illustrates the need to consider whether or not SUDEP victims were genetically susceptible to cardiac arrhythmia when investigating a potential link between SUDEP and treatment with the sodium and potassium channel blocker LTG (21, 32).

Clinical studies, case reports, and animal studies (36, 38–47) have suggested that several mutations in genes encoding cardiac arrhythmia are co-expressed in heart and brain (8). This means that individuals with a cardiac channelopathy may have genuine epileptic seizures, in addition to episodes with cardiac arrhythmia. Furthermore, individuals with convulsive epileptic seizures and a diagnosis of assumed genetic epilepsy may be particularly vulnerable to cardiac arrhythmia when treated with ion channel blockers. Idiopathic epilepsy may be caused by a diversity of mutations in ion channels (48). In addition to IGE, there are also focal genetic epilepsies that are also caused by ion channel mutations (49, 50). However, in clinical practice, it is usually unknown which ion channel mutation(s) may be present in patients with presumed genetic epilepsy, and whether or not such a mutation is co-expressed in heart and brain. Nevertheless, as some individuals with presumed genetic epilepsy will have a mutation predisposing them to cardiac arrhythmia, caution in prescribing treatment with ion-channel blockers, like LTG and CBZ, is reasonable as they could increase the risk of a pro-arrhythmic side effect in this subgroup of individuals with epilepsy. Thus, whether a cardiocerebral channelopathy may have been present in SUDEP victims is essential to take into account when investigating possible associations between CBZ or LTG treatment and SUDEP.

In 2021, the American Food and Drug Administration (FDA) issued an update to LTG labels, with a warning of an increased risk of life-threatening cardiac arrhythmia in individuals with heart disease, including «cardiac channelopathies such as Brugada syndrome» (Lamictal (lamotrigine): Drug Safety Communication - Studies Show Increased Risk of Heart Rhythm Problems in Patients with Heart Disease | FDA). The warning was based on *in vitro* studies and mentioned the sodium channel-blocking properties of LTG; however, inhibition of the cardiac potassium ion current IKr (32) was not noted. In response to this FDA warning, the ILAE and the American Epilepsy Society (AES) Task Force published an *ad hoc* advisory to health professionals to reduce the risk of cardiac arrhythmia in association with LTG treatment (51). Although they reported that they found no clinical studies suggesting a greater risk from LTG compared with other ASM, the studies that were cited had been performed solely on individuals without any known heart conditions (30, 33, 51, 52). In addition, the *ad hoc* advisory did not emphasize that that cardiac channelopathies are among the conditions associated with an increased risk. A 2022 review of studies on SUDEP, sudden cardiac death, and new or worsened ECG abnormalities in individuals that had been treated with LTG (53) reported that none of the identified studies had discussed subgroups of people with epilepsy or included individuals with heart disease. This study concluded that there is insufficient evidence to state whether LTG treatment increases the risk of these outcomes in these individuals, compared with other ASM or placebo (53). Furthermore, a Danish registry-based study (54) found no evidence that LTG elevates the risk of cardiac conduction disorders in individuals without known cardiac morbidity or increases the mortality from all causes in individuals with pre-existing cardiac disorders. The study included individuals with a prescription for LTG, but a diagnosis of epilepsy was not a prerequisite for inclusion. Consequently, the study was not designed to answer the question of whether LTG increases the risk of SUDEP in patients with IGE.

Similarly, there is a paucity of relevant studies addressing the subgroup of females with IGE (Table 1). An ILAE study from 2012 (13) made a separate analysis for female and male SUDEP victims using LTG and found that the OR for SUDEP in females on LTG in monotherapy was 6.6 (CI 0.3–174.9), whereas the OR was 0.4 in males. Compared with controls, the difference was not statistically significant and therefore the authors concluded that there was no elevated risk in this group. However, they did not consider whether the very wide confidence interval could be due to dilution of their material by combining a smaller subgroup with an increased risk (IGE) with a much larger subgroup with focal epilepsy, and thus without an increased risk of SUDEP. It is possible that a difference could have been detected if the subgroup with IGE had been analyzed separately. A study from Norway (12), published at about the same time as the 2012 study (13), provided further evidence suggesting that the risk of SUDEP differed according to patient sex. In concordance with the ILAE study, there were no increase in OR regarding risk in males, but the incidence of SUDEP was five times higher in women that had been treated with LTG than in women that had not been treated with LTG (*p* = 0.007). Furthermore, a significantly higher proportion of the female SUDEP victims were being treated with LTG (58%) than the proportion in controls, matched on age and gender (24.4%) (*p* = 0.038). Whether these findings are mainly due to an inferior efficacy of LTG regarding seizure reduction in this group or whether they reflect a pro-arrhythmic adverse effect in IGE is unclear. However, it could be relevant that the incidence of drug-induced torsade de pointes arrhythmia is up to three times higher in females than in males (56 – 57). The possibility of LTG efficacy being affected by patient sex has not, to our knowledge, been studied.

## Conclusion

The majority of studies exploring a possible association between treatment with CBZ or LTG and SUDEP have concluded that there is no such association but have not taken the possibility of important subgroups in the patient material into consideration. However, for LTG, results from studies that have included subgroup analyses, when taken together, suggest that female patients with IGE may be at an increased risk of SUDEP. This could be because of poorer seizure protection or due to a pro-arrhythmic effect in IGE, or both. For CBZ, only one study analyzed females separately, and did not identify any increase in risk of SUDEP. For future studies, the scientific evidence indicates that it would be of value to conduct a separate analysis for female individuals with IGE. Statistical adjustment for differences in seizure frequency between cases and controls means correction for differences in ASM efficacy. As the efficacy of ASM at reducing seizure frequency is crucial for protection against SUDEP, the usefulness of such as an adjustment is questionable.

## Author contributions

DA: Writing – review & editing, Writing – original draft. ET: Writing – review & editing, Writing – original draft.
